# Transcriptome-wide association study and Mendelian randomization in pancreatic cancer identifies susceptibility genes and causal relationships with type 2 diabetes and venous thromboembolism

**DOI:** 10.1016/j.ebiom.2024.105233

**Published:** 2024-07-12

**Authors:** Marcus C.B. Tan, Chelsea A. Isom, Yangzi Liu, David-Alexandre Trégouët, INVENT Consortium, Sara Lindstrom, Lu Wang, Erin Smith, William Gordon, Astrid Van Hylckama Vlieg, Mariza De Andrade, Jennifer Brody, Jack Pattee, Jeffrey Haessler, Ben Brumpton, Daniel Chasman, Pierre Suchon, Ming-Huei Chen, Constance Turman, Marine Germain, Kerri Wiggins, James MacDonald, Sigrid Braekkan, Sebastian Armasu, Nathan Pankratz, Rabecca Jackson, Jonas Nielsen, Franco Giulianini, Marja Puurunen, Manal Ibrahim, Susan Heckbert, Theo Bammler, Kelly Frazer, Bryan McCauley, Kent Taylor, James Pankow, Alexander Reiner, Maiken Gabrielsen, Jean-François Deleuze, Chris O'Donnell, Jihye Kim, Barbara McKnight, Peter Kraft, John-Bjarne Hansen, Frits Rosendaal, John Heit, Bruce Psaty, Weihong Tang, Charles Kooperberg, Kristian Hveem, Paul Ridker, Pierre-Emmanuel Morange, Andrew Johnson, Christopher Kabrhel, David-Alexandre Trégouët, Nicholas Smith, Lang Wu, Dan Zhou, Eric R. Gamazon

**Affiliations:** a Division of Surgical Oncology and Endocrine Surgery, Section of Surgical Sciences, Vanderbilt University Medical Center, Nashville, TN, USA; b Department of Cell and Developmental Biology, Vanderbilt University, Nashville, TN, USA; c Vanderbilt-Ingram Cancer Center, Nashville, TN, USA; d Herbert Wertheim School of Public Health & Human Longevity Science, University of California, San Diego, San Diego, CA, USA; e Vanderbilt University School of Medicine, Nashville, TN, USA; f INSERM UMR_S 1219, Bordeaux Population Health Research Center, University of Bordeaux, France; g Cancer Epidemiology Division, Population Sciences in the Pacific Program, University of Hawaiʻi Cancer Center, University of Hawaiʻi at Mānoa, Honolulu, HI, USA; h School of Public Health and the Second Affiliated Hospital, Zhejiang University School of Medicine, Hangzhou, China; i The Key Laboratory of Intelligent Preventive Medicine of Zhejiang Province, Hangzhou, China; j Division of Genetic Medicine, Department of Medicine, Vanderbilt University Medical Center, Nashville, TN, USA; k Vanderbilt Genetics Institute, Vanderbilt University Medical Center, Nashville, TN, USA; l Clare Hall, University of Cambridge, Cambridge, United Kingdom

**Keywords:** Genome-wide association study, Models, Genetic, Polymorphism, Single nucleotide, Case-control studies, Mendelian randomization analysis

## Abstract

**Background:**

Two important questions regarding the genetics of pancreatic adenocarcinoma (PDAC) are 1. Which germline genetic variants influence the incidence of this cancer; and 2. Whether PDAC causally predisposes to associated non-malignant phenotypes, such as type 2 diabetes (T2D) and venous thromboembolism (VTE).

**Methods:**

In this study of 8803 patients with PDAC and 67,523 controls, we first performed a large-scale transcriptome-wide association study to investigate the association between genetically determined gene expression in normal pancreas tissue and PDAC risk. Secondly, we used Mendelian Randomization (MR) to analyse the causal relationships among PDAC, T2D (74,124 cases and 824,006 controls) and VTE (30,234 cases and 172,122 controls).

**Findings:**

Sixteen genes showed an association with PDAC risk (FDR <0.10), including six genes not yet reported for PDAC risk (PPIP5K2, TFR2, HNF4G, LRRC10B, PRC1 and FBXL20) and ten previously reported genes (INHBA, SMC2, ABO, PDX1, MTMR6, ACOT2, PGAP3, STARD3, GSDMB, ADAM33). MR provided support for a causal effect of PDAC on T2D using genetic instruments in the HNF4G and PDX1 loci, and unidirectional causality of VTE on PDAC involving the ABO locus (OR 2.12, P < 1e^−7^). No evidence of a causal effect of PDAC on VTE was found.

**Interpretation:**

These analyses identified candidate susceptibility genes and disease relationships for PDAC that warrant further investigation. HNF4G and PDX1 may induce PDAC-associated diabetes, whereas ABO may induce the causative effect of VTE on PDAC.

**Funding:**

10.13039/100000002National Institutes of Health (USA).

**Research in context**

**Evidence before this study**

The diagnosis of pancreatic cancer is commonly associated with the development of a number of non-neoplastic conditions, especially diabetes and venous thromboembolism (VTE). However, few studies have investigated genomic factors associated with the co-occurrence of these diseases.

**Added value of this study**

Our transcriptome-wide association analysis identified six novel susceptibility genes for pancreatic adenocarcinoma. Using Mendelian Randomization, we demonstrated a causal effect of PDAC on T2D using genetic instruments in the HNF4G and PDX1 loci, and unidirectional causality of VTE on PDAC involving the ABO locus.

**Implications of all the available evidence**

The development of diabetes and VTE in patients with PDAC is non-random and influenced by specific genes. Additional human genomic studies as well as mechanistic studies are required to confirm and further explore these findings.

## Introduction

Pancreatic adenocarcinoma (PDAC) is the third leading cause of cancer mortality in the United States, with a five-year survival of just 12%.^1^ Most patients (>80%) present with metastatic or unresectable disease, but there is emerging evidence that screening of individuals at high risk of PDAC identifies cancers at an earlier stage, which in turn is associated with improved survival.^2^ Thus, there is an urgent need to identify germline variants associated with PDAC to expand the pool of high-risk individuals who would benefit from screening.

PDAC is associated with several non-neoplastic diseases, particularly type 2 diabetes (T2D) and venous thromboembolism (VTE). T2D is present in half of PDAC patients.^3^ Meta-analyses have confirmed the association between PDAC and T2D, but proof of causality between them is lacking. Furthermore, the *directionality* of any putative causation (whether diabetes pre-disposes to PDAC, or whether diabetes is a consequence of PDAC) has not been definitively established.^4^ PDAC is also one of the most thrombogenic cancers,^5^ with VTE occurring in approximately 20% of patients.^6^ VTE typically occur within the first 6 months after diagnosis, and are associated with worse prognosis.^7^ Conversely, it is unclear whether VTE plays a causative role in cancer development. Epidemiological studies have shown that 3–5% of patients diagnosed with VTE will have a cancer subsequently diagnosed within a year.^8^ Other studies have shown that there is a persistently increased risk of being diagnosed with a cancer more than a year after the first VTE,^9^^,^^10^ and also if a patient has recurrent VTE.^11^ Mechanistic studies have demonstrated that prothrombotic proteins such as tissue factor, thrombin and fibrinogen, enhance tumor growth and metastasis.^12^, ^13^, ^14^ Crucially, it is not known whether particular germline variants predispose not only to PDAC but also to its associated non-neoplastic phenotypes. This has important clinical implications—for example, *prophylactic* anticoagulation may be indicated in patients with PDAC predicted to be at increased risk of VTE.

Although a number of genome-wide association studies (GWAS), as well as transcriptome-wide association studies (TWAS), have been performed to identify PDAC-associated genes,^15^^,^^16^ only a limited number of genes have been found. Recently, an improved TWAS methodology leveraging the shared regulatory architecture of gene expression across tissues has been developed to boost prediction accuracy, thereby improving the power of the downstream association test. However, given the limitations of conventional TWAS (arising, for example, from SNP instruments showing pervasive horizontal pleiotropy^17^), testing for causal effect remains challenging and further prioritization is needed.^18^ Mendelian Randomization (MR) is a framework to assess causal relationships in observational data, making causal inference in TWAS applications possible. Notably, MR can be performed using only GWAS summary statistics, providing a convenient and potentially powerful approach.^19^^,^^20^

Few MR analyses have examined the causal relationships of PDAC with T2D and VTE. Two MR analyses failed to show a causal effect of PDAC on T2D,^21^^,^^22^ though the second, smaller study demonstrated a small effect of PDAC on new-onset, but not long-standing, diabetes. Cornish et al.^23^ used data from multiple, larger GWAS meta-analyses to examine bidirectional causal relationships between VTE and 18 different cancers, but were unable to show that a causal effect of VTE on cancer, with the possible exception of PDAC. However, all these analyses did not specifically analyze causal relationships at the level of disease-related genes, instead only using SNPs. Therefore, in this study, we sought to fill this specific knowledge gap, by first identifying genes whose genetically determined expression is associated with PDAC using a group of high-accuracy prediction models, then investigating the causative role that these genes play using pleiotropy-robust methods. Finally, using MR, we analyzed the causative role of PDAC on T2D and VTE, and conversely, the causal role of the non-malignant phenotypes on PDAC, using genetics instruments in specific gene loci.

## Methods

The methodology in this study has been reported according to the STREGA guidelines.^24^

### Clinical and genomic data

**We aggregated results from PanScan I-III, PanC4 and BioVU (a total of 8803 PDAC cases and 67,523 controls)** using a fixed-effect meta-analysis. This genome-wide analysis provided an estimate of the overall effect of each genetic variant on PDAC. The summary statistics of GWAS of 8220 patients with PDAC and 6728 controls were obtained from the Pancreatic Cancer Cohort Consortium (PanScan) I, PanScan II, PanScan III, and Pancreatic Cancer Case Control Consortium (PanC4).^25^^,^^26^ These data had been used in our prior publication^15^; genotyping had been performed on the Illumina HumanHap550, 610-Quad, OmniExpress, and OmiExpressExome Arrays, as previously described.^15^ *Additionally*, we leveraged a large, deidentified, single-institution clinical-genomic database, Vanderbilt University's BioVU, which was queried for genotyped adults with pancreatic cancer (ICD9 code 157 and the ICD10 code C25). Genotyping had been performed using the Illumina MEGA^EX^ array. Details regarding patient recruitment, enrolment and genotyping have been previously described.^27^^,^^28^ The medical record was then manually reviewed to confirm the presence and histologic type of pancreatic cancer. From the 91,985 genotyped adults in the database, 1247 individuals with pancreatic cancer were identified by the ICD code-based search. Of these, 749 were confirmed to have pancreatic cancer, with 583 having PDAC. The other 60,795 BioVU participants were used as controls. Study subjects were excluded if they were of non-European ancestry based on genetic estimation.

#### Transcriptome and genome data from the GTEx project

We used transcriptome and genome data from the GTEx v8^29^ to develop genetic imputation models for genes expressed in normal pancreatic tissue. Details of RNA-sequencing experiments, quality control (QC) of the gene expression data, and genomic data have been previously described.^30^^,^^31^

### Statistics

#### Gene expression prediction model training

Gene expression prediction models were trained using pancreatic samples (n = 305) from the GTEx project v8.^29^ The PrediXcan, UTMOST, and JTI frameworks were used to build SNP-based gene expression prediction models. Briefly, the residuals of normalized gene expression levels were used after regressing out covariates, including sex, platform, principal components (PCs), and probabilistic estimation of expression residuals (PEER) factors. SNPs within 1 Mb upstream and downstream of gene bodies were considered as predictor variables for model training. For PrediXcan model training,^32^ the elastic net was applied with five-fold cross validation. For UTMOST,^33^ the model weights were estimated by minimizing the loss function with a LASSO penalty for within-tissue effects, and a group-LASSO penalty for cross-tissue effects. As described in Zhou et al.,^18^ the group penalty term enhances sharing of the information from feature selection across all available tissues. Notably, we modified the original script of UTMOST by using uniform hyper-parameters across different folds to make the hyper-parameters directly comparable.^18^ Performance evaluation in independent datasets confirmed that the modified UTMOST gave an approximately unbiased estimate of prediction performance.^33^ Borrowing information across tissues, JTI (Joint Tissue Imputation) leverages the similarity of regulatory architecture among tissues (here, generated from the DNase I hypersensitivity sites in the promoter region) to boost prediction accuracy.^18^ For each gene, the best prediction performing model (i.e., with the highest prediction accuracy) was selected among PrediXcan, UTMOST, and JTI. The best performing models with r > 0.1 and P < 0.05 for the correlation between observed and predicted expression were defined as imputable genes and were used for downstream analyses.

The associations between predicted expression and risk for PDAC were estimated using summary-statistic-based methods. To further identify putative causal genes, MR-JTI, MR-Egger, and Weighted Median Estimator (WME) were applied. Assuming the presence of widespread horizontal pleiotropy, MR-JTI models the heterogeneity effect for each instrumental variable (IV) and provides an approximately unbiased estimate.

#### Mendelian randomization

To estimate the potential causal effect of mRNA expression on PDAC risk, MR was performed. MR takes germline variants, typically SNPs, as IVs for the exposure of interest (in this case, mRNA expression). The MR approach is based on the following three assumptions: the IVs are associated with exposure; the germline variants are not associated with any confounders; and there is no direct effect of the germline variants on the outcome of interest that is not completely mediated by the exposure. Following published guidelines,^19^ we used independent (R^2^ < 0.01, window size = 250 Kb) exposure-associated SNPs as IVs. Given the genetic architecture of these molecular traits, we mainly considered the *cis* region (1 Mb on both sides of the gene body) to select IVs. To identify potential causal genes, MR-JTI, MR-Egger and WME were implemented. These methods were chosen because each combines data on multiple genetic variants and is robust to certain violations of the IV assumptions.^34^ The 3 methods with their different assumptions provide sensitivity analyses for investigations of causal effects. For all three MR approaches, the effects of genetic variants on gene expression were estimated from the GTEx v8 pancreatic samples. The SNP-PDAC associations were estimated from the fixed-effect meta-analysis.

We further performed MR to infer putative causal associations between VTE and PDAC, as well as T2D and PDAC. WME was applied as a horizontal pleiotropy robust approach. WME provides an unbiased estimation if <50% of the IVs are invalid. MR was performed only if the exposure under test had at least three independent IVs. The GWAS results of VTE (30,234 cases and 172,122 controls) and T2D (74,124 cases and 824,006 controls) were obtained through a collaboration with the INVENT consortium^35^ and Mahajan et al.^36^ (publicly available from https://diagram-consortium.org/), respectively. Once again, study subjects were excluded if they were of non-European ancestry based on genetic estimation. In addition, we asked whether an inferred causal effect was driven by a key gene by using genetic variants near the gene (within 1 Mb) as instrumental variables.

The R package “MendelianRandomization” (0.5.0) was implemented in R 3.6.0 for the MR analyses.

#### Phenome-wide association study

For putative causal genes, phenome-wide association study was performed in BioVU European ancestry samples. Clinical traits were mapped to Phecode as described in Phecode portal (https://phewascatalog.org/phecodes). Trait-associated genes were identified by summary-statistics-based association test using the best prediction performing model as mentioned above.

### Ethics

This work was approved by Vanderbilt University Medical Center's Institutional Review Board: IRB #180823.

### Role of funders

The funders of the study had no role in study design, data collection, data analysis, data interpretation, and writing of the report.

## Results

**Fig. 1:**
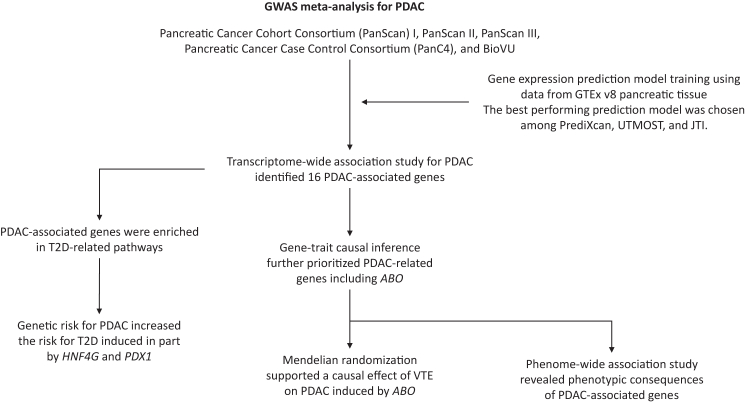
Summary of study cohort. 8803 patients with PDAC and 67523 controls were included. PDAC, pancreatic adenocarcinoma; T2D, type 2 diabetes; VTE, venous thromboembolism; JTI, Joint-Tissue Imputation; UTMOST, unified test for molecular signatures.

The overall study flow is presented in Fig. 1.

### Associations of predicted gene expression in pancreas tissue with PDAC risk

**Fig. 2:**
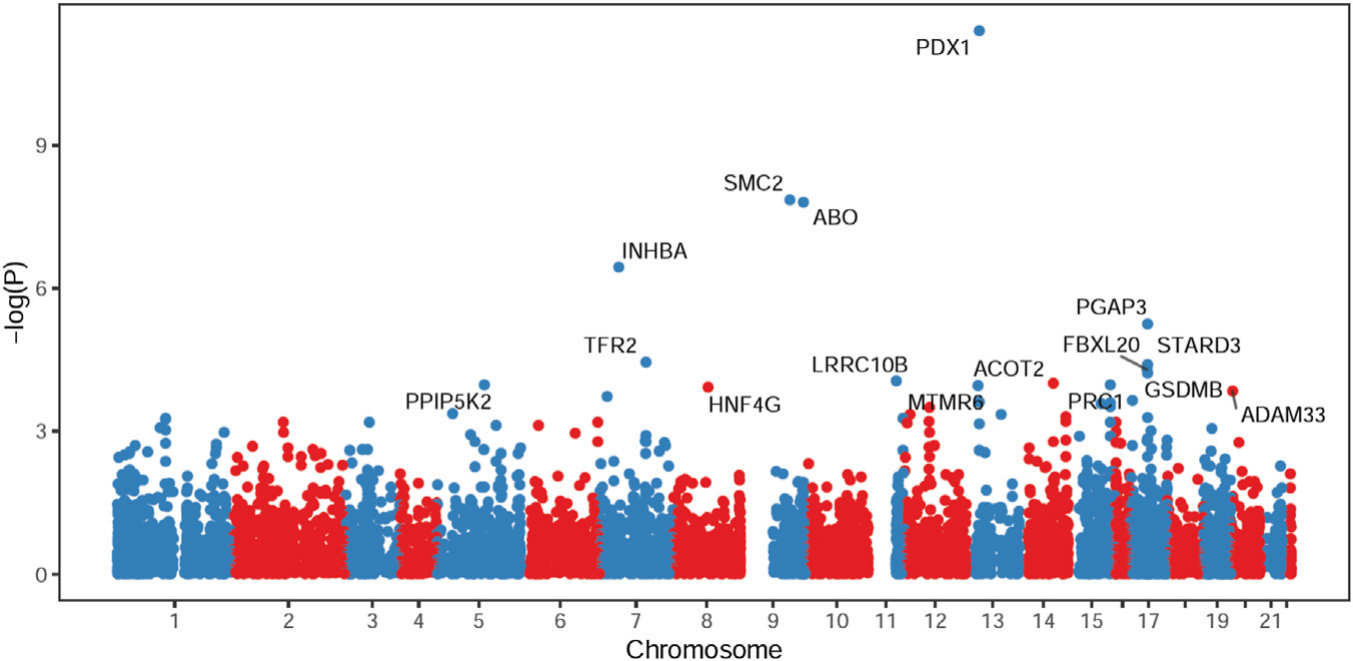
Manhattan plot of association results from the PDAC TWAS. Each dot represents the genetically predicted gene expression of one specific gene by pancreatic tissue prediction models. The x-axis represents the genomic position of the corresponding gene, and the y-axis represents the negative logarithm of the association P-value. The 16 genes that were significantly associated with PDAC at FDR <0.1 have been labelled.

Of the 9952 genes tested, we identified 16 genes whose genetically determined expression was associated with pancreatic cancer risk at FDR <0.10 (Fig. 2).

Of these, 6 have not been reported in previous studies (5q21.1: PPIP5K2; 7q22.1: TFR2; 8q21.13: HNF4G; 11q12.2: LRRC10B; 15q26.1: PRC1; 17q12: FBXL20; Table 1). The 10 remaining genes validate previous reports^15^^,^^16^ (7p14.1: INHBA; 9q31.1: SMC2; 9q34.2: ABO; 13q12.2: PDX1; 13q12.13: MTMR6; 14q24.3: ACOT2; 17q12: PGAP3; 17q12: STARD3; 17q21.1: GSDMB; 20p13: ADAM33; Table 2), with the directionality and magnitude of effect being consistent across the studies, as confirmed by comparison with the TWAS Z-scores from previously published studies.

**Table 1:** PDAC associations for 6 genes that have not previously been reported.

Region	Gene name	R^2^	OR (95% CI)	TWAS Z-score	P	FDR P-value
5q21.1	***PPIP5K2***	0.016	1.587 (1.257, 2.005)	3.877	1.06E-04	7.86E-02
7q22.1	***TFR2***	0.023	1.755 (1.344, 2.290)	4.138	3.51E-05	5.78E-02
8q21.13	***HNF4G***	0.065	1.906 (1.372, 2.648)	3.844	1.21E-04	8.03E-02
11q12.2	***LRRC10B***	0.015	0.543 (0.400, 0.737)	−3.916	8.99E-05	7.86E-02
15q26.1	***PRC1***	0.015	7.389 (2.690, 20.302)	3.879	1.05E-04	7.86E-02
17q12	***FBXL20***	0.014	0.241 (0.121, 0.480)	−4.051	5.10E-05	6.34E-02

**Table 2:** PDAC associations for 10 genes that have been reported in a previous TWAS.

Region	Gene name	R^2^	OR (95% CI)	TWAS Z-score	Range of published TWAS Z-scores^15^^,^^16^	P	FDR P-value
7p14.1	***INHBA***	0.053	0.573 (0.463, 0.710)	−5.091	−5.29 to −5.11	3.56E-07	8.85E-04
9q31.1	***SMC2***	0.025	1.448 (1.274, 1.645)	5.675	4.93 to 5.19	1.39E-08	5.23E-05
9q34.2	***ABO***	0.577	1.147 (1.094, 1.203)	5.653	5.28 to 10.72	1.58E-08	5.23E-05
13q12.2	***PDX1***	0.064	0.476 (0.386, 0.587)	−6.940	−7.18 to −6.54	3.92E-12	3.90E-08
13q12.13	***MTMR6***	0.261	0.830 (0.755, 0.912)	−3.866	−3.82	1.11E-04	7.86E-02
14q24.3	***ACOT2***	0.155	1.175 (1.083, 1.274)	3.895	3.84	9.83E-05	7.86E-02
17q12	***PGAP3***	0.320	1.187 (1.102, 1.278)	4.543	4.43	5.55E-06	1.10E-02
17q12	***STARD3***	0.044	1.697 (1.318, 2.186)	4.104	3.69	4.07E-05	5.78E-02
17q21.1	***GSDMB***	0.060	1.718 (1.319, 2.238)	4.015	3.87	5.95E-05	6.58E-02
20p13	***ADAM33***	0.108	1.323 (1.145, 1.529)	3.802	3.78	1.43E-04	8.91E-02

An association between *higher* genetically determined gene expression and increased PDAC risk was identified for SMC2 (9q31.1), ABO (9q34.2), INHBA (7p14.1), STARD3 (17q12), FBXL20 (17q12), GSDMB (17q21.1), PRC1 (15q26.1), MTMR6 (13q12.13), and HNF4G (8q21.13).

An association between *lower* genetically determined gene expression and increased PDAC risk was identified for PDX1 (13q12.2), PGAP3 (17q12), TFR2 (7q22.1), PPIP5K2 (5q21.1), ACOT2 (14q24.3) and ADAM33 (20p13).

### Enrichment analyses for the 16 TWAS-identified genes associated with PDAC

We performed Gene Ontology (GO)^37^ and pathway enrichment analysis using KEGG^38^ and Reactome^39^ through the DAVID database^40^ of the 16 genes whose genetically predicted expression was associated with PDAC risk. There was enrichment in the pathways involved in beta-cell development (P = 0.02) and maturity onset diabetes of the young (P = 0.02) due to the inclusion of PDX1 and HNF4G.

### Gene-level causal effect inference

**Fig. 3:**
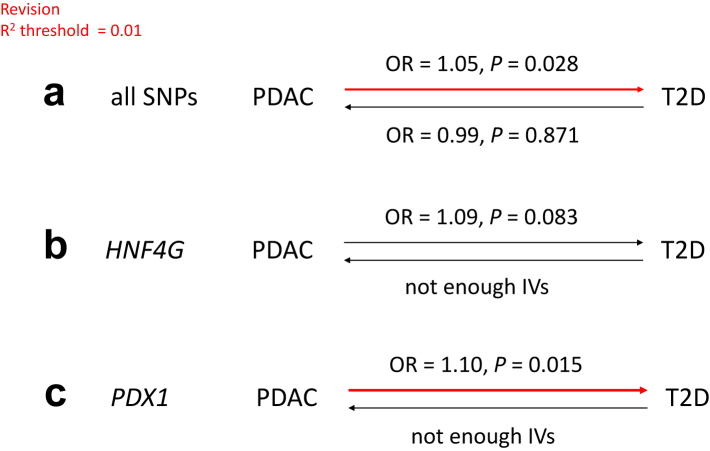
Mendelian Randomization of PDAC and T2D using the WME method. A: MR analysis using all SNPS with P < 5e-8; B and C: MR analysis only using IVs within 1 Mb from HNF4G and PDX1, respectively. IVs, instrumental variables. Red denotes significant causal effect.

MR was performed to determine if there is a causal relationship between PDAC and T2D (Fig. 3). Using the 11 SNPs with P < 5e-8 and *r*^2^ (linkage disequilibrium) threshold of 0.01, we found support for the causal effect of PDAC on T2D (OR = 1.05, P = 0.028), but not vice versa. In addition, individual gene MR (only using IVs within 1 Mb from the gene body) analyses using HNF4G (OR = 1.09, P = 0.083) and PDX1 (OR = 1.10, P = 0.015) also suggested a causal effect of PDAC on T2D. We were not able to estimate the gene-specific causal effect of T2D on PDAC since there were insufficient IVs showing significant association with T2D (P_*FDR*_ < 0.1).

**Fig. 4:**
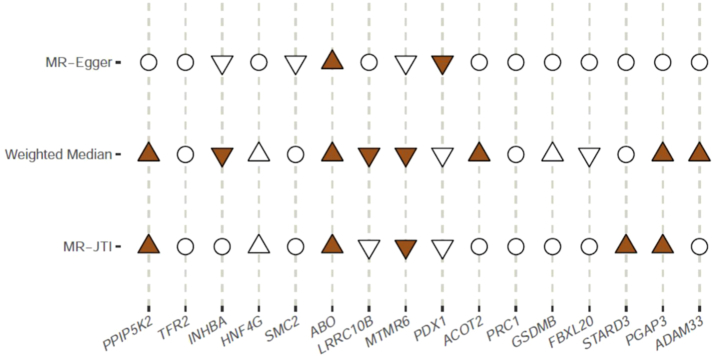
Causal inference testing of the 16 genes whose genetically predicted expression was associated with PDAC risk using three different methods. Hollow circle, hollow triangle, and solid triangle denote results that were non-significant, only nominally significant (P < 0.05), and significant after Bonferroni correction, respectively. Up (positive) and down (negative) triangles indicate the direction of the estimated effect sizes.

For the 16 genes identified from the TWAS analysis, we wanted to assess the causal effect of each gene on the development of PDAC. For sensitivity analysis, this inference was performed using three MR methods that make different assumptions: MR-Egger, WME, and MR-JTI^18^ (Fig. 4). Genes found to be causally associated with PDAC varied by method: using MR-JTI, PPIP5K2, ABO, STARD3 and PGAP3; using WME, PPIP5K2, ABP, ACOT2, PGAP3 and ADAM33, and using MR-Egger, only ABO. Only ABO was significantly associated with PDAC using all three methods.

**Fig. 5:**
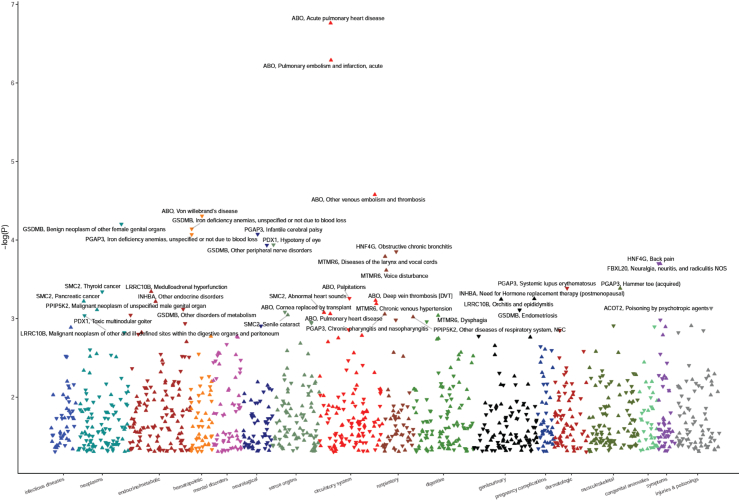
Manhattan plot of association results from the pancreatic cancer PheWAS, using genes which showed significant association in MR for PDAC. Each triangle represents a gene and its related traits. Up (positive) and down (negative) triangles indicate the direction of the estimated effect sizes. The x-axis represents disease categories, and the y-axis represents the negative logarithm of the association P-value.

**Fig. 6:**
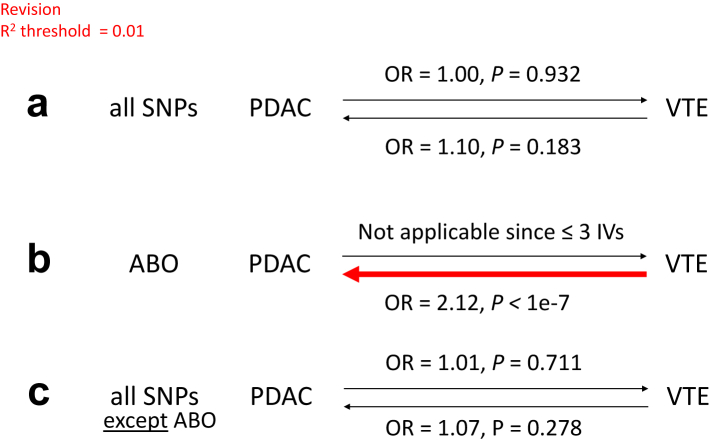
Mendelian randomization of PDAC and VTE using the WME method. A: MR analysis using all SNPs with P < 5e-8; B: MR analysis only using IVs within 1 Mb from ABO. Red denotes significant causal effect.

A PheWAS analysis was then performed using genes in pancreatic tissue which showed significant TWAS association with PDAC with FDR P-value <0.10 (Fig. 5). The strongest association was seen between ABO and its associated VTE traits (e.g., acute pulmonary heart disease, pulmonary embolism and infarction, other venous embolism and thrombosis). Therefore, to further investigate for a causal relationship between PDAC and VTE, MR was performed (Fig. 6). Using all SNPs (P < 5e-8 and *r*^2^ threshold of 0.01), we found no support for PDAC having a causal effect on VTE or vice versa. Strikingly, however, individual MR analysis using genetic instruments in the ABO locus provided support for a causal effect of VTE on PDAC (OR 2.12, P < 1e^−7^, Fig. 6b); a causative effect seemingly purely induced through ABO, as shown by the absence of an effect when the MR was performed excluding ABO (Fig. 6c).

## Discussion

We leveraged the largest available reference dataset for normal pancreas tissue transcriptome, a genetic modeling strategy for gene expression, and a meta-analysis of several large-scale genome-wide association studies of PDAC to evaluate the relationship between genetically determined gene expression in pancreas tissue and PDAC risk. We identified 16 genes whose genetically determined gene expression was associated with PDAC, including six genes not previously reported. Furthermore, using MR, we showed that VTE, induced by ABO, has a strong causal effect on PDAC (rather than vice versa). We also confirmed that PDAC has a causal effect on T2D, induced in part by PDX1 and HNF4G.

Of the genes identified in this study which had not previously been reported as being associated with PDAC, we found mechanistic studies linking all of them to carcinogenesis. TFR2 encodes the transferrin receptor 2, which is part of the hepcidin-regulating iron metabolism pathway. A recent GWAS analysis found that genetic susceptibility related to this pathway is associated with PDAC risk, suggesting a role for iron metabolism in pancreatic carcinogenesis.^41^ FBXL20 encodes F-Box and Leucine Rich Repeat Protein 20, a protein-ubiquitin ligase involved in proteasomal degradation and shown to have roles in progression and chemoresistance in pancreatic,^42^ colorectal^43^ and breast^44^ cancers. PRC1 encodes protein regulator of cytokinesis 1, a substrate of several cyclin-dependent kinases. Increased expression of PRC1 promotes tumor proliferation and cell cycle progression in lung cancer.^45^ PPIP5K2 encodes diphosphoinositol pentakisphosphate kinase 2, an enzyme that regulates a variety of cellular processes, including apoptosis, vesicle trafficking and cytoskeletal dynamics. It has been implicated in the development of colorectal,^46^ cervical^47^ and ovarian^48^ cancers. LRRC10B encodes leucine-rich repeat-containing protein 10B of unknown function, but its paralog LRRC10 has a role in a cardiac development and function. HNF4G encodes the transcription factor hepatocyte nuclear factor 4 gamma, which is upregulated in the setting of SMAD4 deficiency, and has a role in progression and metastasis of PDAC.^49^

There is a long-standing controversy about the relationship between PDAC and T2D. Epidemiological studies have suggested that people older than 50 years with newly diagnosed diabetes have an increased risk of PDAC.^50^, ^51^, ^52^ Even prediabetes appears to confer increased risk of PDAC: a meta-analysis of 2408 patients found that for every 10 mg/dL increase in fasting blood glucose, there is a 14% increase in the incidence of PDAC.^53^ There is evidence supporting a link between *new-onset* T2D and PDAC, particularly in older patients with lower BMI and concomitant weight loss.^50^^,^^54^^,^^55^ Here, the contention is that the cancer in the pancreas is the proximate cause of dysfunction of the endocrine function of the organ, though the mechanisms remain elusive. On the other hand, patients who have had diabetes for years have an increased risk of PDAC, suggesting that more long-standing diabetes is an independent risk factor for the development of PDAC.^56^^,^^57^ A meta-analysis of 44 studies found that the relative risk of PDAC with a duration of diabetes ≥5 years and ≥10 years was 1.58 and 1.50, respectively.^57^ Only two prior studies have used MR to test these epidemiological observations for causal effect. Carreras-Torres et al.^22^ performed the first (unidirectional) MR analysis evaluating this question, and found **no** evidence of a causal effect of PDAC on T2D. Molina-Montes et al.^21^ performed a similar SNP-based MR analysis with the same result, except that there was a causal effect on PDAC on the subset of patients diagnosed with type 2 diabetes in the two years immediately preceding the diagnosis of PDAC (“new-onset” T2D). However, both of these studies had smaller numbers of both patients (7110 and 2018, respectively) and particularly controls (7264 and 1540, respectively). A major confounding factor in these studies is that the true incidence and duration of T2D, whether it be in patients with PDAC or in the general population, is unclear. Screening for diabetes in patients newly diagnosed with PDAC is not recommended in cancer management guidelines, and there is no systematic screening for T2D in healthy individuals. This is further complicated by the increase in the ageing population and obesity, which are also risk factors for both T2D and PDAC. Thus, both the incidence of T2D in PDAC (the “numerator”) and the incidence nationally (the “denominator”) are very hard to estimate. In our study, though we could not determine duration of diabetes, our MR analysis using greater numbers of patients and controls has demonstrated a causal effect of PDAC on T2D (but not vice versa) and extended this work by showing that this effect is partly induced by HNF4G and to a lesser extent by PDX1.

Our MR analysis supports a causal role of ABO in PDAC development. While there is a substantial amount of data associating the ABO gene with PDAC, a *causal* relationship between ABO and PDAC is unclear. The ABO blood group is the most important blood group for determining safety of blood transfusions. Non type O blood type is associated with increased risk of PDAC.^25^^,^^58^^,^^59^ The rs505922 polymorphism in particular has been associated with PDAC.^60^ ABO type is also prognostic in PDAC—those with blood type A have the worst survival; blood type O have the best survival.^61^^,^^62^ However, the mechanisms by which the ABO antigens promote pancreatic carcinogenesis remain poorly defined.

Our phenome-wide scan identified VTE-related ABO traits as the most significant associations with the medical phenome. Patients with PDAC have the highest risk of venous thromboembolism among all cancer patients.^63^ This is due to over-activation of coagulation, which involves a cascade of coagulation proteins and platelets. ABO antigens are expressed on platelet surface membrane glycoproteins. Type O platelets (expressing neither A or B antigens) have lower affinities for von Willebrand factor and slower clot formation. Thus, the increased ABO antigen expression in individuals with non-type O blood may lead to increased platelet aggregation, thereby increasing clotting risk. There is a complex interplay between cancer and coagulation, with tumor cells influencing coagulation in multiple ways.^63^^,^^64^ These effects result from somatic mutations, such as activation of oncogenic *Kras* and inactivation of the *p53* tumor suppressor gene. Conversely, activation of coagulation has been shown to impact pro-oncogenic pathways because coagulation factors are well known to have pleiotropic effects. Thus, germline variants such as ABO that affect the coagulation cascade may play an important role in creating the microenvironment for cellular dysplasia.

A key finding connecting ABO, pancreatic cancer risk and VTE is data showing that two ABO SNPs (rs505922 and rs657152) are associated with both PDAC and cardiocerebrovascular diseases. This is because the unifying pathogenic mechanism underlying both myocardial infarction and stroke is the formation of acute intraluminal clot in the blood vessels of the heart and brain, respectively. A recent meta-analysis^60^ showed that the odds ratios (OR) for the associations between the rs505922 polymorphism and PDAC and cardiocerebrovascular disease were 1.18 (P = 0.001) and 1.36 (P < 0.001), respectively, and for the rs657152 polymorphism, 1.18 (P < 0.001) and 1.54 (P < 0.001), respectively.

To further investigate the intriguing possibility that clotting risk may of itself predispose individuals towards PDAC, we performed bi-directional MR for PDAC and VTE. This demonstrated that there was a strong causal effect of VTE on PDAC induced purely by ABO (OR 2.12, P < 1e^−7^, Fig. 6). Thus, our data support the hypothesis that inherited variation in clotting, particularly through ABO, may increase risk of PDAC. Our findings differ from the recent publication by Cornish et al.,^23^ who did not find any causal effect of VTE on 18 different cancers, with the possible exception of PDAC. They found an association between VTE and PDAC (OR 1.23), which appeared to be induced completely by one SNP, rs687289, located in intron 2 of ABO. Unfortunately, only 45 SNPs (genome-wide) were included in their analysis, with none of the other SNPs being located within 1 Mb of ABO. They therefore concluded that there was insufficient evidence to demonstrate a causal relationship of VTE on PDAC. In contrast, using 16 SNPs within the ABO locus, we found a significant causal effect of VTE on PDAC. Thus, our findings indicate a stronger, more robust conclusion about the causal effect of VTE on PDAC.

There are a number of limitations to this study. First, even though we have identified genes associated with increased risk of PDAC, functional studies to prove their role in disease causation have not been performed and are beyond the scope of this initial work. Second and similarly, proof beyond MR for the causative role of PDAC on diabetes and VTE would rely on animal models of PDAC where diabetes and VTE develop during neoplastic progression, which do not yet exist. Third, confirmation of the causative role of VTE on PDAC via ABO will require a large patient cohort with extremely detailed clinical and genomic annotation—this multi-institutional cohort study is being planned. However, a methodological strength of our study is the addition of a very large number of controls (just over 60,000) from the BioVU dataset. This increased the total number of controls ***ten-fold*** compared to our prior study.^15^ Importantly, the previously identified disease-associated genes remain consistent in their significance and directionality in this new meta-analysis. Finally, though we cannot fully rule out potential pleiotropy, we applied a pleiotropy-robust MR approach, namely weighted median estimator (WME). One of the primary advantages of the weighted median estimator is its robustness to invalid instruments (genetic variants). In Mendelian randomization, some genetic variants used as instruments may not strictly satisfy the instrumental variable assumptions (e.g., they might be pleiotropic, affecting multiple traits). WME can provide a consistent estimate of the causal effect even if up to half of the information comes from invalid instruments.^34^ Another limitation is that WME may be less reliable when the number of IVs is small.^34^ Overall, our work here confirms that ABO has a causative role in the development of PDAC, and our MR analysis supports that ABO induces the effect of VTE on PDAC (vertical pleiotropy). Direct experimental evidence that supports ABO promoting PDAC via thrombotic effects is lacking in the literature and beyond the scope of this current paper. However, variants in ABO determine the activity of the encoded glycosyltransferase, which then affects protein glycosylation. In turn, aberrant glycosylation has been shown to promote both epithelial to mesenchymal transition, an early step in malignant transformation,^65^^,^^66^ *and* hypercoagulability.^67^, ^68^, ^69^, ^70^ Mechanistic studies are required to dissect its role in the inter-relationship between cancer and thrombosis, and thus to determine whether ABO's pleiotropy is truly vertical (as our work suggests) rather than horizontal.

In terms of generalizability of the MR results, the association of PDAC with diabetes is unique among cancers, related to the shared organ of origin of the two diseases. Therefore, it seems unlikely that a causal relationship exists between diabetes and other cancers. On the other hand, it is possible that VTE has a causal effect on a specific set of other cancers via ABO. Epidemiological studies have shown an association between ABO blood groups and risk of cancers of the stomach, esophagus, colon and ovary, as well as the pancreas.^71^ These cancers also have high rates of VTE, though substantially lower than for PDAC,^5^ suggesting that VTE drives a specific spectrum of cancer risk.

In conclusion, this large-scale TWAS of PDAC revealed genes whose genetically predicted gene expression was associated with pancreatic cancer, with MR analyses demonstrating causative roles of PDAC on diabetes, induced in part by HNF4G and PDX1, and a strong causal effect of VTE on PDAC induced by ABO. Further investigation of these relationships will provide new insights into the biology and genetics of PDAC and its associated phenotypes.

## Contributors

MT: conceptualization, formal analysis, investigation, project administration, supervision, writing–original draft, writing–review and editing.

ERG: conceptualization, data curation, formal analysis, investigation, methodology, project administration, resources, supervision, visualization, writing–review and editing.

DZ: data curation, formal analysis, investigation, methodology, validation, visualization, writing–original draft, writing–review and editing.

LW: data curation, investigation, writing–review and editing.

CI: data curation, investigation, writing–review and editing.

LZ: data curation, investigation, writing–review and editing.

INVENT Consortium: data curation, investigation.

All authors have read and approved the final version of this manuscript.

DZ and ERG have accessed and verified the underlying data.

The INVENT Consortium provided GWAS summary statistics from an existing dataset. Dr. Trégouët assisted with revision of the manuscript.

## Data sharing statement

The GWAS data of patients with PDAC and controls of the Pancreatic Cancer Cohort Consortium (PanScan) I, PanScan II, PanScan III, and Pancreatic Cancer Case Control Consortium (PanC4) were accessed from dbGaP at http://www.ncbi.nlm.nih.gov/sites/entrez?db=gap through dbGaP accession phs000206.v5.p3 and phs000648.v1.p1.^25^^,^^26^ Applications were needed to get access to the relevant data. Raw data for the PDAC TWAS is provided in Supplemental File 1. The GWAS results of VTE cohort are not publicly available and were obtained through a collaboration with the INVENT consortium.^35^ The GWAS results for the T2D cohort are publicly available from https://diagram-consortium.org/and Mahajan et al.^36^ The BioVU data can be accessed from https://victr.vumc.org/what-is-biovu/after receiving permission from the review committee. For TWAS, all prediction models used in this study are freely available for download from Zenodo (https://doi.org/10.5281/zenodo.3842289).

## Declaration of interests

L.W. provided consulting service to Pupil Bio Inc. and reviewed manuscripts for *Gastroenterology Report*, not related to this study, and received honorarium. E.R.G. has received funding from the NIH (NIMH R01MH126459 and NIMH RF1MH125933), participated in a NIH study section, and received honorarium, all unrelated to this study. Group members and funding sources for the INVENT Consortium are listed in Supplemental Files 2 and 3, respectively. The other authors declare no potential conflicts of interest.
