# Supplementary material for: Transcriptome-wide association study and Mendelian randomization in pancreatic cancer identifies susceptibility genes and causal relationships with type 2 diabetes and venous thromboembolism
Source: eBioMedicine. 2024 Jul 12;106:105233. doi: 10.1016/j.ebiom.2024.105233 (PMC11284564; doi:10.1016/j.ebiom.2024.105233)
Supplement: Supplemental File 2 [file mmc2.docx]

INVENT Consortium Members

| **First names** | **Surnames** |
| --- | --- |
| Sara | Lindstrom |
| Lu | Wang |
| Erin | Smith |
| William | Gordon |
| Astrid | Van Hylckama Vlieg |
| Mariza | De Andrade |
| Jennifer | Brody |
| Jack | Pattee |
| Jeffrey | Haessler |
| Ben | Brumpton |
| Daniel | Chasman |
| Pierre | Suchon |
| Ming-Huei | Chen |
| Constance | Turman |
| Marine | Germain |
| Kerri | Wiggins |
| James | MacDonald |
| Sigrid | Braekkan |
| Sebastian | Armasu |
| Nathan | Pankratz |
| Rabecca | Jackson |
| Jonas | Nielsen |
| Franco | Giulianini |
| Marja | Puurunen |
| Manal | Ibrahim |
| Susan | Heckbert |
| Theo | Bammler |
| Kelly | Frazer |
| Bryan | McCauley |
| Kent | Taylor |
| James | Pankow |
| Alexander | Reiner |
| Maiken | Gabrielsen |
| Jean-François | Deleuze |
| Chris | O'Donnell |
| Jihye | Kim |
| Barbara | McKnight |
| Peter | Kraft |
| John-Bjarne | Hansen |
| Frits | Rosendaal |
| John | Heit |
| Bruce | Psaty |
| Weihong | Tang |
| Charles | Kooperberg |
| Kristian | Hveem |
| Paul | Ridker |
| Pierre-Emmanuel | Morange |
| Andrew | Johnson |
| Christopher | Kabrhel |
| David-Alexandre | Trégouët |
| Nicholas | Smith |
