# Supplementary material for: Transcriptome-wide association study and Mendelian randomization in pancreatic cancer identifies susceptibility genes and causal relationships with type 2 diabetes and venous thromboembolism
Source: eBioMedicine. 2024 Jul 12;106:105233. doi: 10.1016/j.ebiom.2024.105233 (PMC11284564; doi:10.1016/j.ebiom.2024.105233)
Supplement: Supplemental File 3 [file mmc3.docx]

**FUNDING SOURCES FOR THE INVENT CONSORTIUM**

**Atherosclerosis Risk in Communities (ARIC):** The National Heart, Lung, and Blood Institute (NHLBI) provided support for venous thromboembolism identification via R01 HL059367. The ARIC study is carried out as a collaborative study supported by NHLBI contracts (HHSN268201100005C, HHSN268201100006C, HHSN268201100007C, HHSN268201100008C, HHSN268201100009C, HHSN268201100010C, HHSN268201100011C, and HHSN268201100012C), R01HL087641 and R01HL086694;

National Human Genome Research Institute contract U01HG004402; and National Institutes of Health contract HHSN268200625226C. The authors thank the staff and participants of the ARIC study for their important contributions. Infrastructure was partly supported by Grant Number UL1RR025005, a component of the National Institutes of Health and NIH Roadmap for Medical Research. The authors thank the staff and participants of the ARIC study for their important contributions. Funding support for "Building on GWAS for NHLBl-diseases: the U.S. CHARGE consortium" was provided by the NIH through the American Recovery and Reinvestment Act of 2009 (ARRA) (5RC2HL102419). Jack W. Pattee was supported by NIH T32GM108557.

**Cardiovascular Health Study:** This CHS research was supported by NHLBI contracts HHSN268201200036C, HHSN268200800007C, HHSN268201800001C, N01HC55222, N01HC85079,N01HC85080, N01HC85081,N01HC85082,N01HC85083,N01HC85086;and NHLBI grants U01HL080295, R01HL087652, R01HL105756, R01HL103612, R01HL120393, R01HL085251, and U01HL130114 with additional contribution from the National Institute of Neurological Disorders and Stroke (NINOS). Additional support was provided through R01AG023629 from the National Institute on Aging (NIA). A full list of principal CHS investigators and institutions can be found at CHS-NHLBl.org. The provision of genotyping data was supported in part by the National Center for Advancing Translational Sciences, CTSI grant UL1TR001881, and the National Institute of Diabetes and Digestive and Kidney Disease Diabetes Research Center (DRC) grant DK063491 to the Southern California Diabetes Endocrinology Research Center. The content is solely the responsibility of the authors and does not necessarily represent the official views of the National Institutes of Health.

**CHARGE Hemostasis Working Group:** The Working Group is supported in part by the National Heart, Lung, and Blood Institute (NHLBI) grant HL134894 (NL Smith). Paul S. de Vries was supported by American Heart Association grant number 18CDA34110116. Maria Sabeter­ Lleal is a recipient of a Miguel Servet contract from the Spanish Ministry of Health (ISCIII CP17/00142).

**Early Onset Venous Thrombosis Study (EOVT):** No grants to acknowledge.

**The eMERGE Consortium:** The eMERGE Network was initiated and funded by NHGRI through the following grants: U01HG006828 (Cincinnati Children's Hospital Medical Center/Boston Children's Hospital); U01HG006830 (Children's Hospital of Philadelphia); U01HG006389 (Essentia Institute of Rural Health, Marshfield Clinic Research Foundation and Pennsylvania State University); U01HG006382 (Geisinger Clinic); U01HG006375 (Group Health Cooperative); U01HG006379 (Mayo Clinic); U01HG006380 (Icahn School of Medicine at Mount Sinai); U01HG006388 (Northwestern University); U01HG006378 (Vanderbilt University Medical Center); and U01HG006385 (Vanderbilt University Medical Center serving as the Coordinating Center). *Group Health Cooperative/University of Washington:* Funding support for Alzheimer's Disease Patient Registry (ADPR) and Adult Changes in Thought (ACT) study was provided by a U01 from the National Institute on Aging (Eric B. Larson, **Pl,** U01AG006781 ). A gift from the 3M Corporation was used to expand the ACT cohort. DNA aliquots sufficient for GWAS from ADPR Probable AD cases, who had been enrolled in Genetic Differences in Alzheimer's Cases and Controls (Walter Kukull, Pl, R01 AG007584) and obtained under that grant, were made available to eMERGE without charge. Funding support for genotyping, which was performed at Johns Hopkins University, was provided by the NIH (U01HG004438). Genome-wide association analyses were supported through a Cooperative Agreement from the National Human Genome Research Institute, U01HG004610 (Eric B. Larson, Pl). Assistance with phenotype harmonization and genotype data cleaning was provided by the eMERGE Administrative Coordinating Center (U01HG004603) and the National Center for Biotechnology Information (NCBI). *Marshfield Clinic Research Foundation:* Funding support for the Personalized Medicine Research Project (PMRP) was provided through a cooperative agreement (U01HG004608) with the National Human Genome Research Institute (NHGRI), with additional funding from the National Institute for General Medical Sciences (NIGMS) The samples used for PMRP analyses were obtained with funding from Marshfield Clinic, Health Resources Service Administration Office of Rural Health Policy grant number D1A RH00025, and Wisconsin Department of Commerce Technology Development Fund contract number TDF FYO10718. Funding support for genotyping, which was performed at Johns Hopkins University, was provided by the NIH (U01HG004438). Assistance with phenotype harmonization and genotype data cleaning was provided by the eMERGE Administrative Coordinating Center (U01HG004603) and the National Center for Biotechnology Information (NCBI). *Vanderbilt University:* Funding support for the Vanderbilt Genome-Electronic Records (VGER) project was provided through a cooperative agreement (U01HG004603) with the National Human Genome Research Institute (NHGRI) with additional funding from the National Institute of General Medical Sciences (NIGMS). The dataset and samples used for the VGER analyses were obtained from Vanderbilt University Medical Center's BioVU, which is supported by institutional funding and by the Vanderbilt CTSA grant UL1RR024975 from NCRR/NIH. Funding support for genotyping, which was performed at The Broad Institute, was provided by the NIH (U01HG004424). Assistance with phenotype harmonization and genotype data cleaning was provided by the eMERGE Administrative Coordinating Center (U01HG004603) and the National Center for Biotechnology Information (NCBI). *Geisinger Health System:* Samples and data in this obesity study were provided by the non-alcoholic steatohepatitis (NASH) project. Funding for the NASH project was provided by a grant from the Clinic Research Fund of Geisinger Clinic. Funding support for the genotyping of the NASH cohort was provided by a Geisinger Clinic operating funds and an award from the Clinic Research Fund. Samples and data in this study were provided by the abdominal aortic aneurysm (AAA) project. Funding for the AAA project was provided by a grant from the Clinic Research Fund of Geisinger Clinic. Funding support for the genotyping of the AAA cohort was provided by a Geisinger Clinic operating funds and an award from the Clinic Research Fund.

Samples and data in this study were provided by the Geisinger MyCode Project. Funding for the MyCode project was provided by a grant from Commonwealth of Pennsylvania and the Clinic Research Fund of Geisinger Clinic. Funding support for the genotyping of the MyCode cohort was provided by Geisinger Clinic operating funds and an award from the Clinic Research Fund. *Mount Sinai School of Medicine:* Samples and data used in this study were provided by the Mount Sinai School of Medicine (MSSM) Biobank Project funded by The Charles R. Bronfman Institute for Personalized Medicine (1PM) at Mount Sinai School of Medicine. The Coronary Artery Disease study (1PM BioBank GWAS) is a genome-wide association study funded by the Charles R. Bronfman Institute for Personalized Medicine. The datasets used for the analyses described in this manuscript were obtained from dbGaP at <http://www.ncbi.nlm.nih.gov/gap> through dbGaP accession number phs000888.v1 .p1.

**Framingham Heart Study (FHS):** Framingham Heart Study (FHS) was partially supported by the National Heart, Lung, and Blood lnstitute's (NHLBl's) Framingham Heart Study (Contract No. N01-HC-25195) and its contract with Affymetrix, Inc. for genotyping services (Contract No. N02-HL-6-4278). A portion of this research utilized the Linux Cluster for Genetic Analysis (LinGA-11) funded by the Robert Dawson Evans Endowment of the Department of Medicine at Boston University School of Medicine and Boston Medical Center. Geoffrey Tofler acknowledges funding from the National Institutes of Health (R01-HL-48157). The analyses reflect intellectual input and resource development from the Framingham Heart Study investigators participating in the SNP Health Association Resource (SHARe) project. The views expressed in this manuscript are those of the authors and do not necessarily represent the views of the National Heart, Lung, and Blood Institute; the National Institutes of Health; or the U.S. Department of Health and Human Services.

**The Genotype-Tissue Expression (GTEx) Project** was supported by the Common Fund of the Office of the Director of the National Institutes of Health (commonfund.nih.gov/GTEx). Additional funds were provided by the NCI, NHGRI, NHLBI, NIDA, NIMH, and NINOS. Donors were enrolled at Biospecimen Source Sites funded by NCI\Leidos Biomedical Research, Inc. subcontracts to the National Disease Research Interchange (10XS170), Roswell Park Cancer Institute (1OXS171 ), and Science Care, Inc. (X10S172). The Laboratory, Data Analysis, and Coordinating Center (LDACC) was funded through a contract (HHSN268201000029C) to the The Broad Institute, Inc. Biorepository operations were funded through a Leidos Biomedical Research, Inc. subcontract to Van Andel Research Institute (1OST1035). Additional data repository and project management were provided by Leidos Biomedical Research, lnc.(HHSN261200800001E). The Brain Bank was supported supplements to University of Miami grant DA006227. Statistical Methods development grants were made to the University of Geneva (MH090941 & MH101814), the University of Chicago (MH090951,MH090937, MH101825, & MH101820), the University of North Carolina - Chapel Hill (MH090936), North Carolina State University (MH101819),Harvard University (MH090948), Stanford University (MH101782), Washington University (MH101810), and to the University of Pennsylvania (MH101822). The datasets used for the analyses described in this manuscript were obtained from dbGaP at <http://www.ncbi.nlm.nih.gov/gap> through dbGaP accession number phs000424.v7.p2.

**Health Professional Follow-up Study (HPFS):** See the Nurses' Health Study.

**Heart and Vascular Health (HVH) VTE Study:** The HVH Study was supported by National Heart, Lung, and Blood Institute grants HL43201, HL60739, HL68986, HL73410, HL74745, HL85251, HL95080, and HL121414.

The **JUPITER** trial and its genetic substudy were funded by AstraZeneca.

**Marseille Thrombosis Association Study (MARTHA):** The MARTHA project was supported by grants from the Program Hospitalier de Recherche Clinique. MARTHA genetics research programs are supported and funded by the GenMed LABEX (ANR-10-LBX-0013) and the French INvestigation Network on Venous Thrombo-Embolism (INNOVTE). David-Alexandre Tregouet was financially supported by the "EPIDEMIOM-VTE" Senior Chair from the Initiative of Excellence of the University of Bordeaux.

**Mayo VTE Study:** The research was funded, in part, by grants from the National Institutes of Health, National Heart, Lung, and Blood Institute (HL66216 and HL83141) and the National Human Genome Research Institute (HG04735, HG06379-07, and HG06379-08) and Mayo Foundation.

**Million Veteran Program:** The MVP is funded by the Department of Veterans Affairs Office of Research and Development, Million Veteran Program Grant #MVP000. This publication does not represent the views of the Department of Veterans Affairs or the United States Government. MVP was also supported by three additional Department of Veterans Affairs awards (101- 01BX03340, I01-BX003362, and I01-CX001025). Scott M. Damrauer is supported by the

Veterans Administration (IK2-CX001780). Pradeep Natarajan is supported by the NIH/NHLBI K08HL140203 R01HL142711 and a Hassenfeld Award from the Massachusetts General Hospital.

**Multiple Environmental and Genetic Assessment of risk factors for VT study (MEGA)** The MEGA study was supported by Netherlands Heart Foundation (NHS 98.113), the Dutch Cancer Foundation (RUL 99/1992), the Netherlands Organisation for Scientific Research (912-03-0331 2003), and partially by the Laboratory of Excellence in Medical Genomics (GenMed LABEX ANR-10-LABX-0013). We would like to thank all colleagues from the French Centre National de Genotypage for the genotyping contribution.

**The Nord-Tnzmdelag Health Study (HUNT):** The Nord-Tr0ndelag Health Study (The HUNT Study) is a collaboration between HUNT Research Center (Faculty of Medicine and Health Sciences, NTNU, Norwegian University of Science and Technology), Nord-Tr0ndelag County Council, Central Norway Regional Health Authority, and the Norwegian Institute of Public Health. Ors. Ben Brumpton and Kristian Hveem work in a research unit funded by Stiftelsen Kristian Gerhard Jebsen; Faculty of Medicine and Health Sciences, NTNU; The Liaison Committee for education, research and innovation in Central Norway; and the Joint Research Committee between St. Olavs Hospital and the Faculty of Medicine and Health Sciences, NTNU. The genotyping in HUNT was financed by the National Institute of Health (NIH); University of Michigan; The Research Council of Norway; The Liaison Committee for education, research and innovation in Central Norway; and the Joint Research Committee between St.

Olavs Hospital and the Faculty of Medicine and Health Sciences, NTNU. The K.G. Jebsen Center for Genetic Epidemiology is financed by Stiftelsen Kristian Gerhard Jebsen, Faculty of Medicine and Health Sciences Norwegian University of Science and Technology (NTNU) and the Liaison Committee for education, research and innovation in Central Norway. Dr. Brumpton

is financed by the Medical Research Council Integrative Epidemiology Unit at the University of Bristol which is supported by the Medical Research Council and the University of Bristol [MC_UU_12013/1].

**Nurse's Health Study (NHS and NHS-II) and Health Professionals Follow Up Study:** The work for the current study was funded by grants from the NIH: P01CA87969, R01CA49449, R01HL034594,R01HL088521, R01CA50385,R01CA67262, P01CA055075, R01HL35464, R01HL116854.

**The Troms0 Study:** The Troms0 Study was supported by an independent grant from Stiftelsen Kristian Gerhard Jebsen in Norway (J.B.H.).

**UK Biobank:** This research has been conducted using the UK Biobank Resource under Application number 25298.

**The Women's Genome Health Study (WGHS):** The WGHS is supported by the National Heart, Lung, and Blood Institute (HL043851 and HL080467) and the National Cancer Institute (CA047988 and UM1CA182913), with funding for genotyping provided by Amgen.

**The Women's Health Initiative (WHI):** The WHI program is funded by the National Heart, Lung, and Blood Institute, National Institutes of Health, U.S. Department of Health and Human Services through contracts HHSN268201100046C, HHSN268201100001C, HHSN268201100002C, HHSN268201100003C, HHSN268201100004C and HHSN271201100004C.
